# Therapeutic advances in acute myeloid leukemia: from LSD1 blockade to PROTAC-based strategies

**DOI:** 10.1097/MS9.0000000000004650

**Published:** 2026-01-09

**Authors:** Sarum A. Khan, Muhammad A. Qamar, Muhammad T. Feroze

**Affiliations:** a Department of Medicine, Wah Medical College, Wah Cantt, Pakistan; b Department of Medicine, Spinghar Medical University, Kabul, Afghanistan

**Keywords:** acute myeloid leukemia (AML), bomedemstat, E3 ligase, epigenetic dysregulation, GFL1B, hematological toxicity, immature blood cells, LSD1 inhibitors, lysine-specific demethylase (LSD1), proteolysis targeting chimeras (PROTAC), repressor complex

## Abstract

Acute myeloid leukemia (AML) is a fast-progressing blood cancer marked by immature myeloid cells and poor survival rates. Most AML subtypes resist standard treatments, especially in older adults, highlighting the need for new targeted therapies. A hallmark of AML is LSD1 overexpression, which blocks blood cell maturation. Though some LSD1 inhibitors can trigger differentiation, their use is limited by toxicity and incomplete target inhibition.

PROTAC degraders like MS9117 offer a promising solution by eliminating LSD1. This halts leukemia growth, promotes robust cell differentiation, and restores sensitivity to agents like all-trans retinoic acid, benefits not seen with traditional inhibitors. PROTACs can also target previously undruggable proteins, such as transcription factors, potentially enabling lower doses and fewer side effects. Several PROTAC therapies are now in AML trials. LSD1-targeted degradation is promising, but further research is needed to confirm its safety, overcome resistance, and identify optimal drug combinations for AML treatment.

*To The Editor,*

This manuscript is in accordance with the TITAN checklist guidelines 2025^[1]^.

Acute myeloid leukemia (AML) is a highly lethal hematological malignancy characterized by the uncontrolled proliferation of immature blood cells in the bone marrow and peripheral blood. The global incidence of AML nearly doubled from 79 372 cases in 1990 to 144 645 in 2021, with a disproportionate impact on men and older adults^[2]^. Despite aggressive treatment regimens, the 5-year survival rate remains below 50%, and treatment-related toxicities are considerable, underscoring the urgent need for safer and more effective therapeutic strategies. Acute promyelocytic leukemia (APL), a distinct AML subtype, is curable with all-trans retinoic acid (ATRA) and arsenic trioxide; however, non-APL AML is resistant to these agents. Standard therapies, including intensive chemotherapy and stem cell transplantation, induce remission in up to 80% of younger, fit patients, yet 5-year survival remains below 50%, with even poorer outcomes observed in older or less fit individuals^[3]^. These challenges highlight the necessity for targeted, biology-driven interventions, such as LSD1-directed therapies. Epigenetic dysregulation is a hallmark of AML, with lysine-specific demethylase 1A (LSD1/KDM1A) overexpressed in approximately 60% of cases^[4]^. LSD1 demethylates histone H3K4 and H3K9 and has been recognized since 2004 as a key regulator of reversible histone modifications. Overexpression of LSD1 is also observed in various solid tumors, where it impedes differentiation, promotes proliferation and migration, and correlates with poor prognosis^[5]^. In normal hematopoiesis, LSD1 forms a repressor complex with the transcription factor GFI1B to suppress differentiation genes. CRISPR-suppressor screening in AML has demonstrated that LSD1 disruption abrogates this interaction, leading to derepression and activation of differentiation gene enhancers, such as IRF8, thereby reducing leukemic self-renewal^[6]^. Several small-molecule LSD1 inhibitors, including tranylcypromine derivatives, ORY-1001 (ladademstat), bomedemstat, INCB059872, MC2580, and DDP-38 003, have entered early-phase clinical trials and promote blood cell differentiation. Notably, MC2580 and DDP-38 003 act by downregulating GSE1^[7,8]^. Hematological toxicity, especially low platelets, remains dose-limiting; reversible inhibitors reduce but do not eliminate this risk^[9]^.

Proteolysis-targeting chimeras (PROTACs) are heterobifunctional molecules composed of one region that binds the target protein, another that recruits an E3 ligase, and a linker that connects these domains. By bringing the target protein and the E3 ligase into proximity, PROTACs induce ubiquitination and subsequent proteasomal degradation. The LSD1 degrader MS9117 demonstrates more pronounced anti-leukemia effects compared to traditional inhibitors. By recruiting CRBN and activating the ubiquitin-proteasome system, MS9117 rapidly and durably eliminates LSD1, including its scaffolding functions. This is a significant advantage, as these scaffolding functions maintain the differentiation block and are not affected by inhibitors. MS9117 is effective at lower doses, eliminates LSD1, suppresses cellular growth and colony formation, and induces robust blood cell differentiation. Additionally, it restores ATRA sensitivity, resulting in synergistic effects not observed with inhibitors alone^[10]^.

PROTACs enable targeting proteins that were previously difficult to drug, including transcription factors, scaffold proteins, and mutant oncoproteins. One PROTAC molecule can destroy multiple copies of a target, reducing drug load, side effects, and off-target activity. Over 3270 PROTACs have been developed, with six antileukemic ones in early trials: CDK6-targeting BSJ-03-123 and CP-10, FLT3-targeting TL13-117/149 and PROTAC-67, and MDM2-targeting MD-224 and MS-3227. Effectiveness depends on linker design, E3 ligase selection, and ternary complex stability, and even weak ligand binding can still lead to substantial degradation. PROTACs offer a selective, catalytic approach that outperforms traditional inhibitors and holds promise for AML and targets such as CDK6 and FLT3^[11]^.

LSD1 degradation is a promising therapeutic avenue, but clinical translation remains incomplete. PROTAC degraders, such as MS9117, overcome many of the limitations of catalytic inhibitors, yet broader testing across AML subtypes is needed. Future studies should assess long-term safety, resistance mechanisms, and rational combinations, especially with retinoids, to establish whether LSD1 degraders can be incorporated into standard AML therapy.

## Data Availability

All data used are accessible.
